# COVID‐19 vaccine acceptability among healthcare workers in Ethiopia: Do we practice what we preach?

**DOI:** 10.1111/tmi.13742

**Published:** 2022-03-20

**Authors:** Daniel Yilma, Rezika Mohammed, Seid Getahun Abdela, Wendemagegn Enbiale, Fasil Seifu, Myrthe Pareyn, Laurens Liesenborghs, Johan van Griensven, Saskia van Henten

**Affiliations:** ^1^ Department of Internal Medicine Jimma University Jimma Ethiopia; ^2^ Jimma University Clinical trial Unit Jimma University Jimma Ethiopia; ^3^ Department of Internal Medicine University of Gondar Gondar Ethiopia; ^4^ Department of Internal Medicine Wollo University Dessie Ethiopia; ^5^ Department of Dermatology Bahir Dar University Bahir Dar Ethiopia; ^6^ Department of Dermatology Amsterdam Institute for Infection and Immunity Academic Medical Centre Amsterdam Netherlands; ^7^ Department of General Surgery Arba Minch University Arba Minch Ethiopia; ^8^ Department of Clinical Sciences Institute of Tropical Medicine Antwerpen Belgium

**Keywords:** COVID‐19, healthcare workers, SARS‐CoV‐2, vaccine acceptability, vaccine hesitancy

## Abstract

**Objective:**

We assessed healthcare workers (HCWs) COVID‐19 vaccine acceptability in Ethiopia.

**Methods:**

We carried out a cross‐sectional survey from February to April 2021 in HCWs from five teaching hospitals. HCWs were selected using convenient sampling, and data were collected through a survey link. Descriptive analysis and mixed‐effect logistic regression were performed. A total of 1,314 HCWs participated in the study.

**Results:**

We found that 25.5% (*n* = 332) of the HCWs would not accept a COVID‐19 vaccine and 20.2% (*n* = 264) were not willing to recommend COVID‐19 vaccination to others. Factors associated with vaccine non‐acceptance were female sex (AOR = 1.8; 95% CI: 1.3–2.5), the perception that vaccines are unsafe (AOR = 15.0; 95% CI: 8.7–25.9), not considering COVID‐19 as health risk (AOR = 4.4; 95% CI: 2.0–9.5) and being unconcerned about contracting COVID‐19 at work (AOR = 3.5; 95% CI: 1.5–8.4). Physicians were more willing to accept vaccination than other HCWs. Higher vaccine acceptability was also noted with increasing age. Participants most often indicated safety concerns as the determining factor on their decision to get vaccinated or not.

**Conclusion:**

Overall, a quarter of HCWs would not accept a COVID‐19 vaccine. Communications and training should address vaccine safety concerns. Additionally, emphasis should be given to showing current and future impact of COVID‐19 on the personal, public and country level unless control efforts are improved. Interventions aimed to increase vaccine uptake should focus their efforts on younger and non‐physician HCWs.

## INTRODUCTION

Africa is heavily affected by COVID‐19 and has the highest global mortality rate in critically ill COVID‐19 patients [1]. As countries often have insufficient resources to provide adequate support for critically ill COVID‐19 patients [1], prevention measures are key to reduce mortality and morbidity. In sub‐Saharan Africa (SSA), Ethiopia has seen relatively high numbers of COVID‐19 patients. Since the first case of COVID‐19 was reported in Ethiopia on 13 March 2020, the Ethiopian Ministry of Health has been designing and implementing various preventive strategies to control the transmission and reduce mortality [2].

Long‐term control of COVID‐19 will depend on the effective roll‐out of the available vaccines against SARS‐CoV‐2. However, vaccine hesitancy, defined as refusal of vaccines despite their availability, impede vaccination programmes all over the globe. In 2014, the WHO Strategic Advisory Group of Experts on Immunization put vaccine hesitancy as a top 10 global health threat, and with the COVID‐19 pandemic, this topic has become even more pressing [3]. Vaccine hesitancy is determined by several factors, including perceptions on effectiveness and safety of the vaccine, trust in health systems and policymakers and perceived risk of the vaccine‐preventable disease [4]. In addition, a low‐risk perception of COVID‐19 infection has also been associated with higher COVID‐19 vaccine hesitancy [5].

Healthcare workers (HCWs) are vital in providing guidance and recommendations to patients and the wider community about vaccination, which includes giving correct information on the risks and benefits of the vaccine. As they are crucial players in vaccine delivery and have a large impact on vaccine acceptability in the community, it is important to know their attitudes towards vaccination. Studies showed that recommendation by the HCWs was crucial in the community willingness to get vaccinatesd against COVID‐19 [6, 7]. Moreover, HCWs are an important first target group for vaccination because they are at high risk of infection.

Ethiopia introduced COVID‐19 vaccination on 13 March 2021, and several vaccines (e.g. Johnson & Johnson, AstraZeneca, Pfizer, Sinopharma, Sinovac) have been made available [8, 9, 10, 11]. A national deployment and vaccination plan was developed following the WHO prioritising roadmap, with prioritised vaccination for frontline health workers and support staff, elderly with underlying conditions and other high‐risk groups [8]. A total of 10,894,936 vaccine doses have been administered in Ethiopia by end of 2021 which is <10% of the total population [12]. Global vaccine inequity combined with vaccine hesitancy has contributed to low COVID‐19 coverage in Ethiopia. Therefore, we aimed to assess COVID‐19 vaccine acceptability among HCW and identify drivers of vaccine non‐acceptance in Ethiopia.

## METHODS

### Study design and setting

We conducted a cross‐sectional survey at five tertiary teaching hospitals in Ethiopia: University of Gondar specialized Hospital, University of Gondar; Tibebe Ghion specialized Hospital, Bahir Dar University; Arba Minch General Hospital, Arba Minch University; Jimma Medical Centre, Jimma University; and Dessie Comprehensive Specialized Hospital, Wollo University. Three sites (Gondar, Bahir Dar and Dessie) are in north Ethiopia and Arba Minch and Jimma are found in southern and southwest Ethiopia respectively. Vaccine was not yet available at the sites during data collection except in Arba Minch and last 2 weeks of data collection in Dessie.

### Population, sample size and sampling procedure

Healthcare workers working in the teaching hospitals were asked to participate in the study. Convenience sampling with stratification by profession was used to collect data of 255 participants at each site. We calculated the sample size based on the acceptability of COVID‐19 vaccination of 27% among HCWs in the Democratic Republic of the Congo [13], using a 3% marginal error, 98% confidence level and 5% non‐response rate, which led to an overall sample size of 1249.

### Data collection

We collected data from 1 February 2021 to 16 April 2021 using a questionnaire developed on the KoBo Toolbox application (www.kobotoolbox.org). Prior to data collection, the questionnaire was tested for consistency, and data collectors were trained. The questionnaire was sent via a link by email to participants, or participants were requested to complete using the application installed at the mobile phone of the data collectors. Anonymity and confidentiality of participants were assured by not collecting personal identifying information.

### Variables

The answer to the question: ‘If a COVID‐19 vaccine is proven safe and effective and is available, will you get vaccinated?’ was used to assess vaccine acceptability. Participants who answered that they will definitely not or probably not get the vaccine were grouped as having vaccine non‐acceptance, while those who answered probably or definitely will get the vaccine were grouped as willing to accept COVID‐19 vaccination. Similarly, the response to the question ‘Would you recommend your patients to get vaccinated for COVID‐19 was used to assess whether participants would recommend the vaccine to others’. Questions to assess attitude and practice of respondents were designed using a Likert scale. Participants were requested to give a score from 1 to 5 to indicate the importance of the information on safety, cost, previous COVID‐19 infection and information about COVID‐19 for deciding to get vaccinated or not; 1 for least important and 5 most important. We also assessed the frequency participants used specific sources of information (social media, official international sites, official governmental sites, news media and scientific journals and conferences) for obtaining information regarding COVID‐19 using a score from 1 (not at all) to 5 (very frequent).

### Statistical analysis

Descriptive statistics were used to describe the sociodemographic characteristics of health workers per site, using numbers and proportions and medians and interquartile (IQR) ranges. As data were collected at some sites before vaccination was available in Ethiopia and in some other sites after vaccination was started, we used a mixed‐effect logistic regression model to identify factors associated with vaccine non‐acceptance controlling site as random effect. We first included all variables with *p*‐value <0.1 in univariate analysis, and we performed backward regression, retaining variables in the final model if the *p*‐value was below 0.05. STATA version number 14.2 was used for analysis.

### Ethics approval

Ethical approvals from all the institutional review boards of the participating institutions were granted before the study started. Each participant gave consent before filling the questionnaire.

## RESULTS

A total of 1314 HCWs from the five teaching hospitals Jimma Medical Centre (*n* = 253), University of Gondar Specialized Hospital (*n* = 288), Tibebe Ghion Specialized Hospital (*n* = 255), Dessie Comprehensive Specialized Hospital (*n* = 256) and Arba Minch General Hospital (*n* = 262) were included in the study. The median age of the HCWs was 29 years (IQR: 27, 33) and 42.5% (*n* = 555) were females. The HCWs had a median experience of 5 years (IQR: 3, 9) in the health sector. Nurses accounted for 38.1% (*n* = 501) of the participants and physicians 21.2% (*n* = 279) (Table 1). Among the participants, 44.4% (*n* = 582) received prior training on COVID‐19, 28.2% (*n* = 368) had been or were taking care of COVID‐19 patients and 31.1% (*n* = 406) had been diagnosed previously with COVID‐19.

**TABLE 1:** Baseline characteristics of healthcare workers by site

	Bahir Dar (*N* = 255)	Dessie (*N* = 256)	Gondar (*N* = 288)	Jimma (*N* = 253)	Arba Minch (*N* = 262)	Total (*N* = 1314)
Age, years	28 (26, 30)	30 (28, 35)	30 (27, 35)	28 (26, 31)	30 (27, 34)	29 (27, 33)
Sex, male	195 (76.8)	153 (60)	167 (59)	111 (43.8)	126 (48.1)	752 (57.5)
Religion						
Orthodox Christian	238 (93.3)	165 (64.5)	227 (80.2)	129 (51.4)	131 (50)	890 (68.1)
Protestant Christian	7 (2.8)	7 (2.7)	21 (7.4)	69 (27.5)	125 (47.7)	229 (17.5)
Muslim	10 (3.9)	84 (32.8)	35 (12.4)	50 (19.9)	5 (1.9)	184 (14.1)
Other	0 (0)	0 (0)	0 (0)	3 (1.2)	1 (0.4)	4 (0.3)
Occupation						
Nurse	62 (24.3)	140 (54.7)	97 (33.7)	112 (44.3)	90 (34.4)	501 (38.1)
Physician	118 (46.3)	36 (14.1)	34 (11.8)	56 (22.1)	35 (13.4)	279 (21.2)
Lab technician	23 (9)	32 (12.5)	29 (10.1)	21 (8.3)	19 (7.3)	124 (9.4)
Midwife	17 (6.7)	7 (2.7)	33 (11.5)	14 (5.5)	39 (14.9)	110 (8.4)
Pharmacist	16 (6.3)	6 (2.3)	29 (10.1)	17 (6.7)	19 (7.3)	87 (6.6)
Cleaner	0 (0)	7 (2.7)	23 (8)	24 (9.5)	4 (1.5)	81 (6.2)
Others	19 (14.4)	28 (21.2)	43 (32.6)	9 (6.8)	33 (25)	132 (10)
Highest level of education						
Specialist/doctorate	100 (39.2)	19 (7.4)	32 (11.2)	55 (21.7)	15 (5.7)	221 (16.8)
Master	17 (6.7)	17 (6.6)	96 (33.6)	9 (3.6)	2 (0.8)	141 (10.7)
Degree	131 (51.4)	176 (68.8)	122 (42.7)	151 (59.7)	143 (54.6)	723 (55.1)
Diploma	7 (2.8)	38 (14.8)	30 (10.5)	16 (6.3)	75 (28.6)	166 (12.6)
Certificate	0 (0)	6 (2.3)	6 (2.1)	22 (8.7)	27 (10.3)	61 (4.7)
Monthly income						
>10,000 birr	70 (27.6)	36 (14.1)	53 (18.9)	45 (17.7)	31 (11.8)	235 (18)
5000–10,000 birr	123 (48.4)	183 (71.5)	191 (68.2)	134 (53)	129 (49.2)	760 (58.2)
1000–5000 birr	61 (24)	37 (14.5)	36 (12.9)	74 (29.3)	102 (38.9)	310 (23.8)
Year working in health sector	4 (2, 6)	6 (3, 10)	6 (3, 10)	5 (2, 7)	6 (4, 10)	5 (3, 9)

### COVID‐19 vaccine hesitancy among HCWs

**FIGURE 1:**
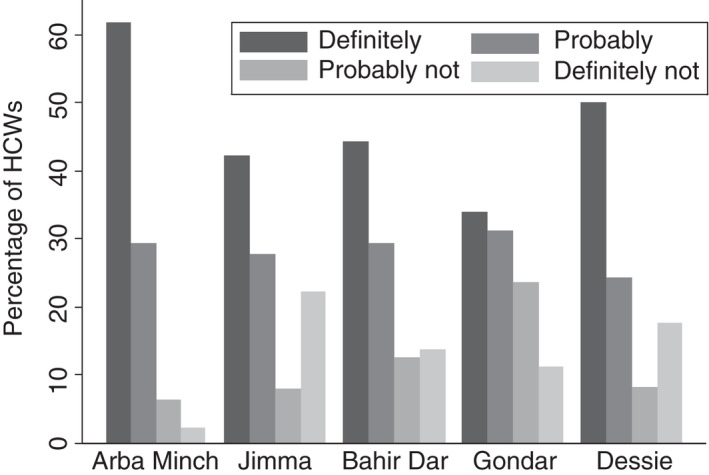
COVID‐19 vaccine acceptibility among healthcare workers (HCWs) by site in Ethiopia. Figure shows the response to the question ‘If a COVID‐19 vaccine is proven safe and effective and is available, Will you get vaccinated?’. The different responses are shown in different shades, and results are shown stratified by site

**FIGURE 2:**
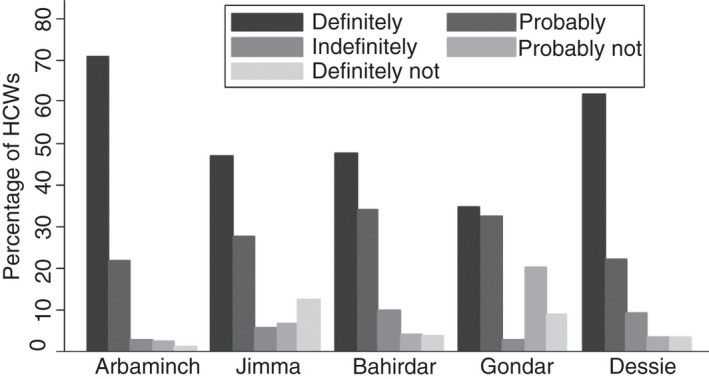
Healthcare workers' (HCWs) COVID‐19 vaccination recommendation by site in Ethiopia. Figure shows the response to the question ‘Would you recommend your patients to get vaccinated for COVID‐19?’. The different responses are shown in different shades, and results are shown stratified by site

One‐fourth (25.2%, *n* = 332) of the HCWs were unwilling to receive COVID‐19 vaccine and one‐fifth (20.2%, *n* = 264) of HCWs were not willing to recommend others to get vaccinated. Vaccine non‐acceptance ranged from 8.8% (*n* = 23) in Arba Minch to 34.7% in Gondar (Figure 1). Similarly, the non‐willingness to recommend vaccination was highest in Gondar (32.6%) and lowest in Arba Minch (7.3%) (Figure 2).

### HCW attitudes towards COVID‐19 vaccination

The majority of the participants (66.9%; *n* = 871) indicated that the country that developed the COVID‐19 vaccine mattered for their decision to get vaccinated. In addition, 45.1% (*n* = 587) of the HCWs thought that a COVID‐19 vaccine can be used before it is shown to be effective and safe and 7.7% (*n* = 101) of the HCWs generally perceived vaccines to be unsafe. Among the HCWs, only 40.3% (*n* = 528) felt adequately protected from COVID‐19 during professional activities. The majority of HCWs were concerned about COVID‐19 in general (91.9%; *n* = 1204) and also worried about contracting COVID‐19 at work (93.7%; (*n* = 1225)) (Table 2).

**TABLE 2:** Factors associated with vaccine non‐acceptance among healthcare workers in Ethiopia

	*N* (%)	COR (95% CI)^a^	*p* ^a^	AOR (95% CI)^b^	*p* ^b^
Age, years	1312 (100)	0.96 (0.94, 0.99)	0.001	0.97 (0.95, 0.99)	0.020
Female gender	555 (42.5)	2.24 (1.72, 2.93)	<0.001	1.81 (1.3, 2.5)	<0.001
Religion					
Protestant Christian	229 (17.5)	Reference		–	–
Orthodox Christian	890 (68.1)	1.65 (1.07, 2.57)	0.02	–	–
Muslim	184 (14.1)	1.63 (0.96, 2.76)	0.07	–	–
Other	4 (0.3)	1.45 (0.14, 15.1)	0.76	–	–
Occupation					
Physician	279 (21.2)	Reference		Reference	
Nurse	501 (38.1)	7.96 (4.8, 13.2)	<0.001	4.72 (2.71, 8.22)	<0.001
Lab technician	124 (9.4)	5.93 (3.22, 10.89)	<0.001	4.32 (2.2, 8.46)	<0.001
Midwife	110 (8.4)	4.9 (2.54, 9.46)	<0.001	2.15(1, 4.61)	0.049
Pharmacist	87 (6.6)	3.13 (1.52, 6.44)	0.002	2.35 (1.07, 5.21)	0.034
Cleaner	81 (6.2)	8.5 (4.27, 16.95)	<0.001	2.56 (1.14, 5.73)	0.022
Others	132 (10)	6.68 (3.64, 12.28)	<0.001	5.52 (2.84, 10.73)	<0.001
Highest level of education					
Specialist/doctorate	221 (16.8)	Reference		–	–
Master	141 (10.7)	5.18 (2.65, 10.12)	<0.001	–	–
Degree	723 (55.1)	7.46 (4.25, 13.08)	<0.001	–	–
Diploma	166 (12.6)	12.06 (6.19, 23.47)	<0.001	–	–
Certificate	61 (4.7)	10.07 (4.43, 22.87)	<0.001	–	–
Monthly income					
>10,000 birr	235 (18)	Reference		–	–
5000–10,000 birr	760 (58.2)	3.06 (1.97, 4.74)	<0.001	–	–
1000–5000 birr	310 (23.8)	4.77 (2.94, 7.74)	<0.001	–	–
Year working in health sector (IQR)	1294 (100)	0.98 (0.96, 1.01)	0.15	–	–
Previous diagnosis with COVID‐19	900 (68.9)	0.94 (0.7, 1.27)	0.69	–	–
No previous use of other vaccination, other than childhood vaccination	249 (19.1)	1.44 (1.06, 1.97)	0.02	–	–
Not taking care of COVID‐19 patients	935 (71.8)	1.24 (0.92, 1.68)	0.17	–	–
Has not taken COVID‐19 training	729 (55.6)	1.68 (1.28, 2.19)	<0.001	–	–
Country that developed the vaccine does not matter	431 (33.1)	0.84 (0.63, 1.12)	0.212	–	–
General attitude towards vaccines					
Safe	952 (72.4)	Reference		Reference	
Undecided	261 (19.9)	3.4 (2.48, 4.66)	<0.001	2.4 (1.68, 3.43)	<0.001
Unsafe	101 (7.7)	17.18 (10.36, 28.51)	<0.001	15.03 (8.73, 25.87)	<0.001
Thinks COVID‐19 vaccines will be used before shown to be effective and safe					
Not likely	569 (43.4)	Reference		–	–
Indifferent/do not know	155 (11.8)	1.42 (0.95, 2.13)	0.09	–	–
likely	587 (44.8)	1.03 (0.76, 1.38)	0.87	–	–
General concern about COVID‐19					
Concerned	1204 (91.9)	Reference		Reference	
Undecided/do not know	54 (4.1)	2.76 (1.57, 4.85)	<0.001	1.96 (0.98, 3.92)	0.06
Not concerned	53 (4)	9.13 (4.83, 17.24)	<0.001	4.38 (2.01, 9.51)	<0.001
Worry about contracting COVID‐19 during work					
Yes	1225 (93.7)	Reference		Reference	
Undecided/do not know	42 9 (3.2)	4.62 (2.41, 8.87)	<0.001	1.57 (0.73, 3.38)	0.25
No	41 (3.1)	7.39 (3.71, 14.71)	<0.001	3.53 (1.48, 8.43)	0.004
Feeling protected from COVID‐19 during professional activities					
Unprotected	732 (55.8)	Reference		–	–
Undecided/do not know	51 (3.9)	1.5 (0.8, 2.82)	0.21	–	–
Protected	528 (40.3)	0.95 (0.72, 1.25)	0.73	–	–

Abbreviations: AOR, adjusted odds ratio; CI, confidence interval; COR, crude odds ratio; *N*, number; *p*, *p*‐value.

^a^ All values are in mixed model controlled for random effect of site.

^b^ All values are in mixed model controlled for fixed effects for variables in column and random effect for site.

The HCWs scored safety as most important factor affecting their choice of getting vaccinated (score 4 and 5) (89.3%, *n* = 1165), followed by cost (55.8%, *n* = 723), previous COVID‐19 infection (23.4%, *n* = 296) and insufficient information about COVID‐19 (28.5%, *n* = 366) (Figure S1a–d). Some HCWs also mentioned other factors like information on the effectiveness of the vaccine, trust in vaccine production, local studies or evidence about the vaccine, safety issues for pregnant and lactating mothers and conspiracy theories.

### Sources of information regarding COVID‐19

News media (72.6% *n* = 953), social media (68.8% *n* = 896), official governmental sites (63.1% *n* = 828), official international sites (48.9%, *n* = 642) and scientific journals and conferences (16.7% *n* = 214) were the most frequently used sources of information (score 4 or 5) (Figure S2a–e).

### Factors associated with COVID‐19 vaccine hesitancy

Groups that significantly associated with vaccine non–acceptance in the multivariate analysis were females (AOR = 1.8; 95% CI: 1.3, 2.5), those who consider vaccines to be generally unsafe (AOR = 15.0; 95% CI: 8.7, 25.9) or are unsure about vaccine safety (AOR = 2.4; 95% CI:1.7, 3.4), those unconcerned about COVID‐19 (AOR = 4.4; 95% CI: 2.0, 9.5) and those unconcerned about contracting COVID‐19 at work (AOR = 3.5; 95% CI: 1.5, 8.4). HCWs that were older were more likely to accept (AOR = 0.97; 95% CI: 0.95, 0.99), and all categories of healthcare workers were less likely to accept than physicians (*p* < 0.05) (Table 2).

### HCWs prioritisation of the COVID‐19 vaccine

Most HCWs prioritised HCWs as the first group (66.4%) to receive the vaccine. People with comorbidities (40.1%), the elderly (36.4%), working adults (46.5%) and children (48.3%) were considered as second, third, fourth and fifth priority groups respectively (Figure S3).

## DISCUSSION

In our survey, one in four Ethiopian HCWs was not willing to receive a COVID‐19 vaccine and one in five was not willing to recommend to others to take the vaccine. These numbers are in line with those found in other surveys among HCWs in different parts of the world, where COVID‐19 vaccine hesitancy ranged from 21% to 62% [13, 14, 15, 16, 17, 18, 19, 20, 21, 22]. A survey conducted by the African Centre for Disease Control and Prevention in 15 African countries found similar results, with 4 of 5 adults in the general population willing to accept the COVID‐19 vaccine if deemed safe and effective [23]. However, the study showed significant variation across countries in Africa in willingness to accept the vaccine: ranging from 59% in the Democratic Republic of Congo to 94% in Ethiopia [23]. In contrast, other similar studies in Ethiopia reported a much lower vaccine acceptance rate that ranged from 31.4% to 45.5% in adults [24, 25, 26]. Moreover, a recent study among Ethiopian HCWs indicated that nearly two‐thirds of them were reluctant to receive COVID‐19 vaccine [27]. The relatively high vaccine acceptance rate we found in our study may be due to our study population. Most included HCWs were active in teaching hospitals where training and education are common, and therefore awareness on the importance of vaccination might be higher.

We identified several groups with higher vaccine non‐acceptance: HCWs who were female, younger, non‐physicians, unconcerned about COVID‐19 and the risk of getting infected at work. These findings are similar to those from other studies [13, 19, 21, 22, 28, 29, 30]. The higher vaccine non‐acceptance in females and younger age is consistently reported in other studies and attributed to their low‐risk perception of COVID‐19 and additionally the higher vaccine safety concern especially in females [13, 19, 21, 28, 29, 31, 32]. The low‐risk perception in some of HCWs in our study may be related to the relatively high recovery and low mortality rates observed in most Ethiopian hospitals. This may also be the reason why we noted a higher vaccine non‐acceptance among HCWs who were unconcerned about either COVID‐19 in general or contracting COVID‐19 at work. The higher vaccine acceptance in physicians compared with other HCWs may be attributed to differences in the education level as reported in other studies [13, 15, 19, 21, 31]. Educational campaigns should be targeted towards groups that had lower vaccine acceptance such as nurses, young HCWs and females. It is also crucial to show the impact of COVID‐19 at personal, public and country level in trainings and communications, especially for groups that do not see direct immediate benefit of getting vaccinated.

HCWs rated vaccine safety as the most important factor to decide whether to get the vaccine. Moreover, we found that HCWs who considered vaccines to be unsafe were less likely to accept vaccination. Safety concerns, which may be related to the speedy development of COVID‐19 vaccines, have been reported as an important factor for vaccine hesitancy among HCWs across the world [15, 17, 21, 22, 28, 29, 33]. Another study in Ethiopia among HCWs also showed that lack of belief in COVID‐19 vaccine benefits, lack of trust in the government, lack of trust in science to produce safe and effective vaccines and concerns about vaccine safety were associated with COVID‐19 vaccine hesitancy [27]. We also found that almost half of the HCWs thought that the COVID‐19 vaccine would be used before it was proven to be effective and safe and that about 8% perceived vaccines in general as unsafe. This may indicate a lack of trust in vaccine development and production or lack of adequate and accurate information among HCWs.

The HCWs also reported that information regarding vaccine development, effectiveness, safety and vaccine conspiracy theories guides them in their decision to receive the vaccine or not. The overabundance of information and misinformation (infodemics) at global scale during the COVID‐19 pandemic may negatively influence HCWs willingness to receive the vaccination [34]. WHO suggested developing trusted sources, fact‐checking and responding to misinformation through dedicated dashboards as strategies to manage infodemics [34]. We found that news media, social media and governmental official websites were the most frequently used sources of information in order of frequency regarding acquiring knowledge on COVID‐19 by HCWs in Ethiopia. Therefore, the government should explore the best use of news and social media to foster trust in vaccination by passing accurate information regarding vaccine safety and effectiveness and tackling anti‐vaccine campaigns and misinformation.

We performed a multicentre study covering a large part of Ethiopia and with a relatively large sample size. The study has several limitations. We used convenience sampling which may result in sampling bias. Some sites collected data before and others after vaccination had started, which may have an impact on the attitude of HCWs. This may be the reason for the higher vaccine acceptance noted among HCWs in Arba Minch compared with other sites. While we pre‐tested our questionnaire before use, it has not been formally validated nor was it guided by a behavioural framework model. Due to social desirability bias and the conduct of our study at teaching university hospitals, our findings may be an underestimation of true vaccine non‐acceptance in HCWs.

## CONCLUSION

Our findings showed that a quarter of HCWs would not accept a COVID‐19 vaccine, and one‐fifth was not willing to recommend others to get vaccinated in Ethiopia. Vaccine non‐acceptance was higher in female, younger, non‐physician HCWs, those who generally perceived vaccines as unsafe, and those who were unconcerned about COVID‐19 in general or about contracting COVID‐19 at work. Based on our findings, we recommend the following:

- Addressing HCWs' COVID‐19 safety concerns is crucial to improve vaccine acceptability.
- Communications and training should emphasise the current and future impact that COVID‐19 may have on the personal, public and country level unless control efforts are improved.
- Interventions aimed to increase vaccine uptake should focus their efforts on younger and non‐physician HCWs.

## Supporting information

Fig S1‐S3
