# Supplementary material for: COVID‐19 vaccine acceptability among healthcare workers in Ethiopia: Do we practice what we preach?
Source: Trop Med Int Health. 2022 Mar 20;27(4):418–25. doi: 10.1111/tmi.13742 (PMC9115514; doi:10.1111/tmi.13742)

**Supplementary figure 1A-D** – HCWs perception on level of importance of different factors for their decision towards COVID-19 vaccine acceptance (5-High importance, 1 least important)

1A. Safety 1B. Cost


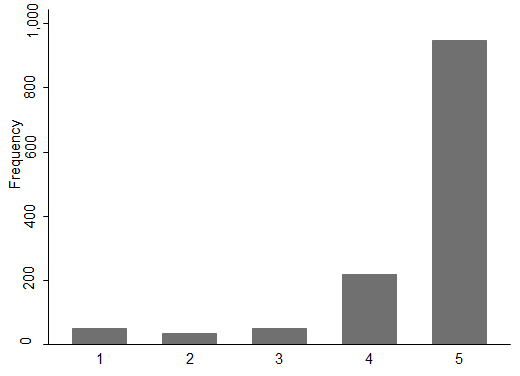

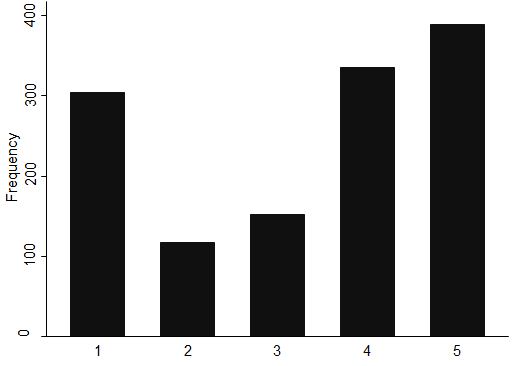


1C. Previous COVID-19 infection 1D. Insufficient information


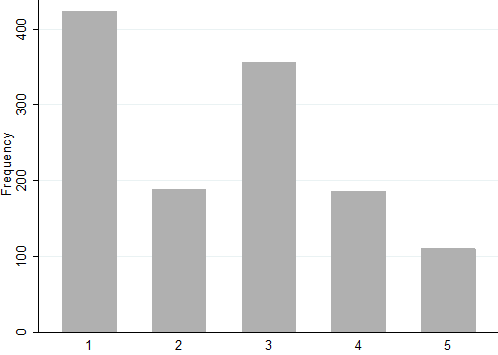

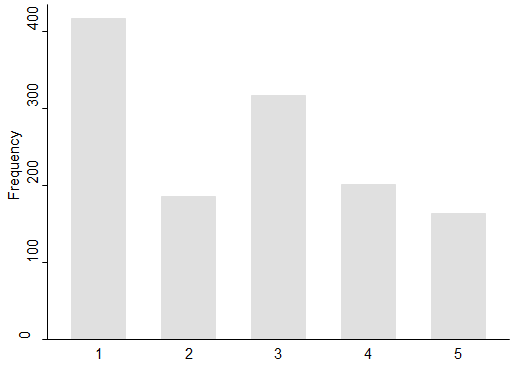


**Supplementary figure 2A-E**. HCWs source of information regarding COVID-19 (5-most frequently used, 1 least frequently used)

2A. Official International site 2B. Official governmental site 2C. News media


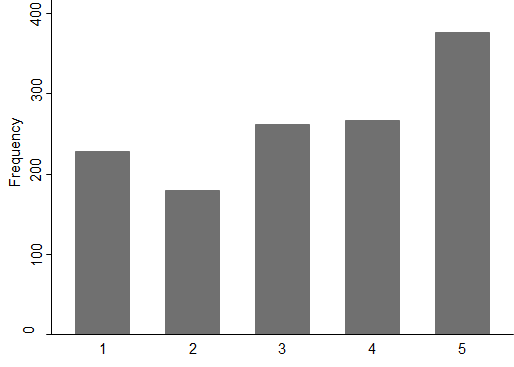

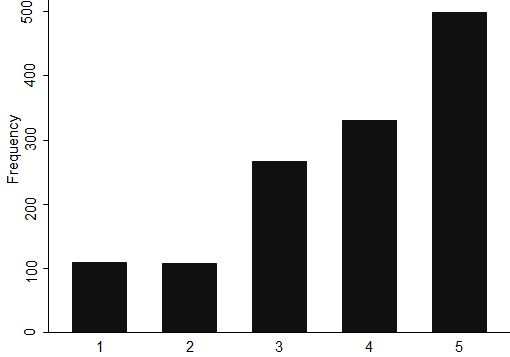

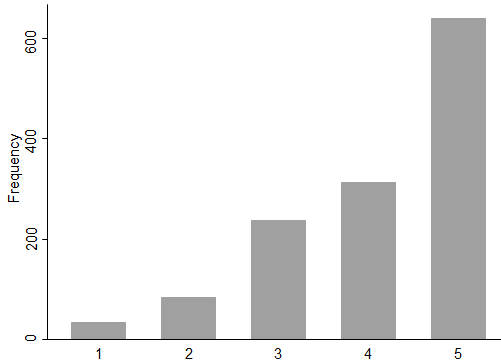


2D. Social media 2E. Scientific journals and conferences


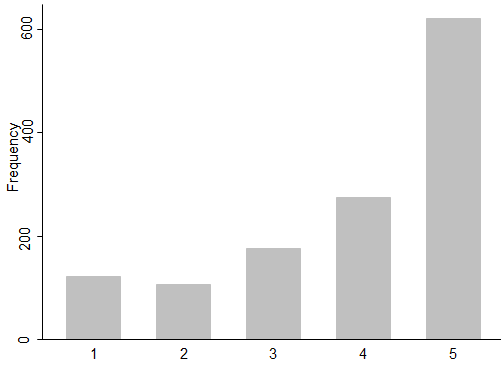

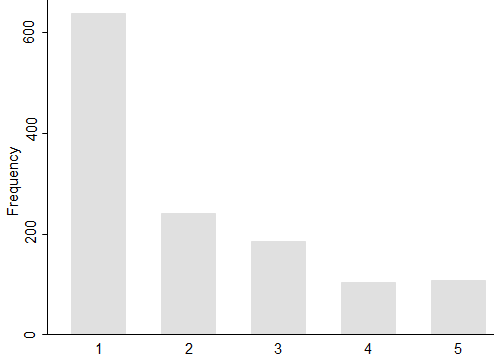


**Supplementary figure 3A-E**. HCWs consideration for prioritization COVID-19 vaccination in Ethiopia

3A. Groups that get first priority 3B. Groups that get second priority


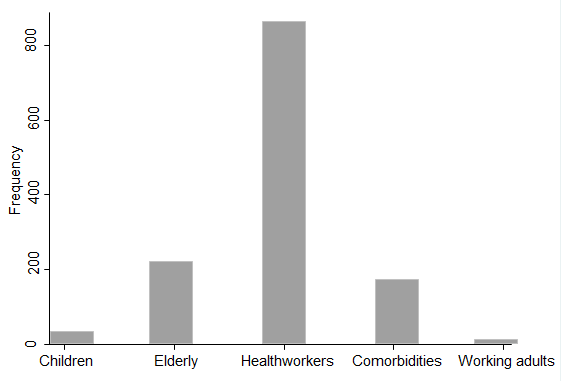

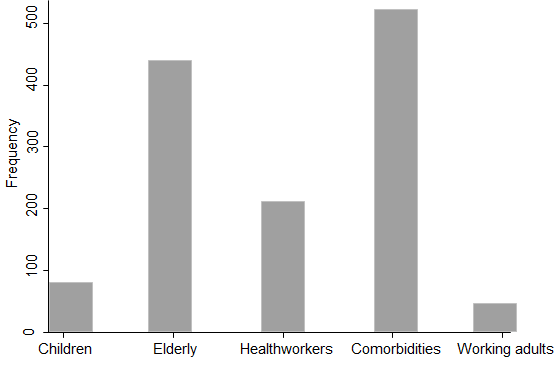


3C. Groups that get third priority 3D. Groups that get fourth priority 3E. Groups that get fifth priority


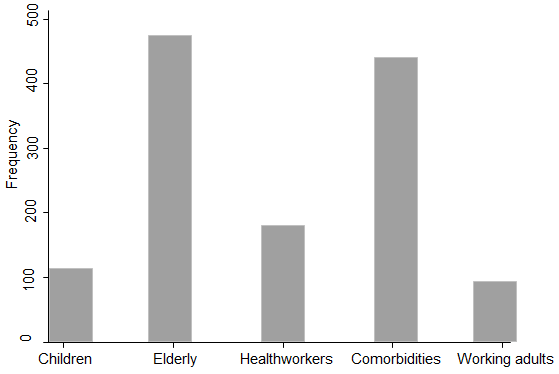

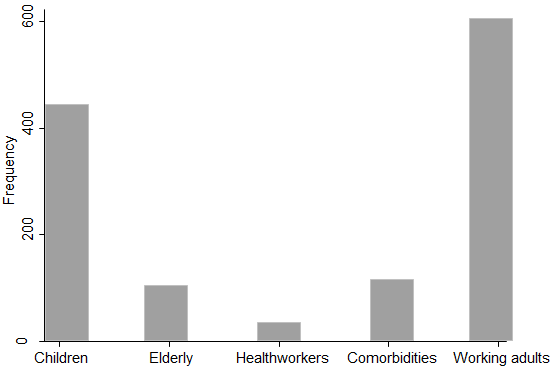

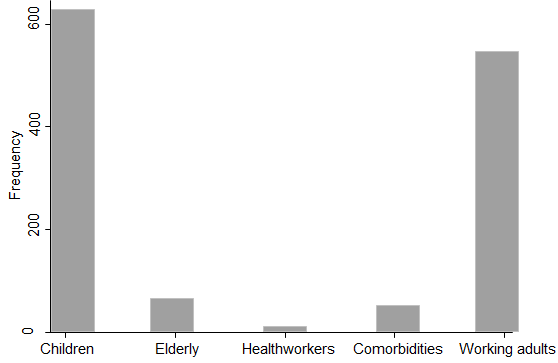

Supplement: Supplementary file 1 — Fig S1‐S3 [file TMI-27-418-s001.docx]
